# PROTOCOL: Effectiveness of public‐private partnerships on educational access and quality of primary and secondary schooling in low‐ and middle‐income countries: A systematic review

**DOI:** 10.1002/cl2.1385

**Published:** 2024-03-05

**Authors:** Sajid Ali, Sadia Muzaffar Bhutta, Sohail Ahmad, Aisha Naz Ansari, Afaq Ahmed, Yasir Qadir

**Affiliations:** ^1^ Institute for Educational Development Aga Khan University Karachi Sindh Pakistan

## Abstract

This protocol serves as a guiding source for a Campbell registered systematic review aiming to assess the effectiveness of Public‐Private Partnerships (PPPs) in education in Low‐ and Middle‐Income Countries (LMICs). The primary question for this review is: What is known about the impact of PPPs on improving access (i.e., enrolment, attendance, drop‐out, and completion) and quality (i.e., students’ learning outcomes and teachers’ teaching practices) of primary and secondary schooling in LMICs? Using a predefined search query, we will retrieve studies from various databases including Scopus, Campbell Library, EBSCO Education Research Complete, NBER, Dissertation and Theses Global (ProQuest), along with other sources of grey literature. Covidence will be used for screening and extraction. A bias assessment tool will be used for the included studies. A standardised mean difference (SMD) effect size of Hedges’ g will be calculated for the outcome variables using RevMan.

This review will provide updated evidence about the impact of PPP in school education across LMICs, which will be instrumental for policy and practice level decisions for education improvement.

## 1 BACKGROUND

The concept of public‐private partnerships (PPPs) represents a blended governance mechanism between different state and non‐state stakeholders over a range of development initiatives (Robertson et al., 2012). Historically, the public sector has been considered the main provider of public services. In doing so, the state has been considered responsible for developing policy, building infrastructure, managing, and executing operations – in short, being responsible for all aspects of public service delivery (Li & Akintoye, 2003). For example, in the health sector, a state, in general, was supposed to develop health policies, build hospitals, employ medical staff, arrange medications, and look after all aspects related to service delivery to ensure that the public has access to reasonable health services.

However, the state, mainly in LMICs, encounters various challenges in delivering public services efficiently. These challenges stem from factors such as bureaucratic complexity, political involvement, limited budgets, and other constraints, resulting in discrepancies between the demand for high‐quality services and the availability of adequate resources to meet this demand. To bridge this gap, private entities have taken the initiative to provide services independently (Cuéllar‐Marchelli, 2003), which has also been critiqued due to inequitable access to the services as well as the state's legitimacy of authority.

In response, the concept of public‐private partnerships (PPPs) has emerged as a new governance model. PPPs involve the shared responsibilities between the public and private sectors, intending to enhance service quality and expand access (Casady et al., 2019). There exist multiple arrangements of partnerships between the public and the private sector, hence it is important to view PPP arrangements on a continuum that encompasses pure private and public service provisions on opposite ends, with various forms of PPPs in between. The theory of change underlying the PPPs in education, particularly in LMICs, suggests that through utilising the financial capacity of the public sector and the management capacity of the private sector, better schooling for all children is ensured.

The partnerships between the public and the private sectors have attracted both positive and negative reviews. On the positive side, the scholars highlight that PPPs have enabled the state to plug the large gaps that continue to exist in terms of quality, access, governance, and financing of education (LaRocque, 2008). Through PPPs the state and the private sector can combine their efforts to provide more choices and better managed schools, which can generate positive competition and improve quality (Barrera‐Osorio et al., 2009). In some specific contexts in developing countries like Pakistan and Uganda the PPPs interventions through subsidy and vouchers have been found useful in increasing access to education and cost‐effective for government (Barrera‐Osorio & Filmer, 2016; Crawfurd, 2017).

On the negative side, scholars highlight that in PPP arrangements the private sector privileges the interests of private entities in education policy to sustain their market‐driven agenda (Robertson & Verger, 2012) and promote segmented education system exacerbating inequities (Patrinos et al., 2009).

Considering these contrasting arguments for and against the PPPs in education, recent systematic reviews are inconclusive, mixed, and provide insufficient basis for causal claims (Aslam et al., 2017; Crawfurd & Hares, 2021). The effect of PPPs depends on the context, the specific sector, and the design of the program. Therefore, to understand the effectiveness of PPP holistically, a broader view of existing evidence based on different models need to be provided. In particular, it has been suggested that more research evidence needs to be analysed to see whether PPPs has been able to improve learning outcomes and access to education.

It is imperative that we find out how and to what extent the PPPs are useful in improving the access to and quality of school education in LMICs. Hence, there is a need to carry out a systematic review to explore what kinds of effects PPPs have on educational access and quality in LMICs to understand their usefulness.

## 2 PROBLEM

Given the limited resources the states, particularly in LMICs, have been struggling to ensure access to quality education for its population, particularly to the marginalised groups. The PPPs were used as a viable and politically more acceptable mechanism to overcome the large gaps that continue to exist in terms of quality, access, governance, and financing of education (LaRocque, 2008). In the early 1990s, a significant increase in the PPPs in education was observed particularly in the UK and the USA (Davies & Hentschke, 2006; Fitz & Beers, 2002; Li & Akintoye, 2003). The World Bank (1997) underlined that the state should not be the exclusive provider of goods and services; instead, it should concentrate on overseeing the delivery of services ensuring their quality and equity. The report signified a reconceptualization of the role of the state, which was to become a regulator rather than the provider of public services – a shift referred to as movement from government to governance (Rosenau, 2000). The processes of globalisation are considered responsible for this alteration in the national sphere of authority (Ali, 2017); whereby the state has to renegotiate its authority with several national and international actors due to their rising influence (Scholte, 2005). Bob Jessop (2002) concluded that globalisation has made the state ‘hollowed out’.

The development of charter schools in the USA and academy schools in the UK during 1990s and 2000s were used as case/evidence for extending the PPPs in education to other developing and developed nations (LaRocque, 2008). In the developing countries, which often suffer from weak management of schools, low educational financing, and poor quality of educational provision, the idea of PPPs was promoted as a viable option to overcome these challenges. Thus, several projects have been introduced during 2000s under different development projects to promote different models of PPPs in lower‐middle‐income countries (Snilstveit et al., 2015; Verger, 2012).

Consequently, international organisations took part in funding the projects promoting public‐private partnerships to efficiently manage public services. Kirkemann and Appelquist (2008) argued that large international organizations, such as the World Bank (WB), Organization for Economic Cooperation and Development (OECD), United Nations International Children's Emergency Fund (UNICEF), United States Agency for International Development (USAID), and Department for International Development (DfID), had prioritised funding for partnerships to achieve the Millennium Development Goals (MDGs) by the late 2000s (Roberston & Verger, 2012) and later Sustainable Development Goals (SDGs).

However, understanding the potential of PPPs in education to comprehensively address the issues of quality, access, governance and financing is complex. It has been inferred that while PPPs in education can increase access to education through more affordable means to the government (Barrera‐Osorio et al., 2017; Crawfurd, 2017), the reliance on PPPs to improve quality education is not always viable (Crawfurd & Hares, 2021). Despite concerns, developing countries are expanding the PPPs in education to tackle diverse educational issues ranging from access to resource constraints to governance and quality.

It is important and urgent that we find out how and to what extent the PPPs is useful or not in minimizing the issues related to access, quality, governance, and financing of education (Mundy & Menashy, 2012). Therefore, this review specifically focuses on the effectiveness of PPPs in providing access to and quality of education in low‐ and middle‐income countries.

## 3 INTERVENTION

The PPP in education is a programme where the public and the private sectors collaborate to achieve the common objectives of ensuring quality education. In PPP, the public (means the state) acts mainly as a financial provider and takes the role of monitor/regulator, whereas the private entity mainly provides services in terms of teachers’ development, provision of resources, running of schools, etc. There are a number of models based on the role and nature of public and private entities in the programme. Examples include (Patrinos et al., 2009; Verger, 2012):

1. Educational Management Organisation (EMO) – the state provide finance and look after accountability whereas a private entity looks after the management of school;
2. Voucher school programme – the state provides finance to the parents, enabling them to choose right school for their children;
3. Foundation‐assisted schools – the state provides financial and technical support to the partner private schools to enrol students;
4. Adopt a school model – philanthropic organization or individual adopts a government school and tries to improve it;
5. Charter schools – these schools are publicly funded but operate independently of the traditional public school system.

The public‐private partnership is an educational intervention that is practiced in both developed and developing countries to ensure better access, quality, and governance. This review focuses on all the above mentioned types of PPPs and tries to synthesize their effectiveness, in terms of improved access and quality in education, based on the available evidence from LMICs.

## 4 THE RATIONALE FOR CONDUCTING THIS REVIEW

We are witnessing the rise of PPPs across the globe including the LMICs. They are often promoted to improve the management and quality of public schooling through the involvement of the private sector. However, there are several shortcomings in PPPs that critics have pointed out, including that PPP in education: is promoting privatisation (Savas & Savas, 2000; Bayliss & Van Waeyenberge, 2018), is furthering inequity (Bano, 2008; PCE, 2015), and is promoted despite lack of conclusive independent evidence (Levin & Kaddar, 2011; Verger et al., 2017). It is also interesting to note that a disproportionate literature in support of PPPs is produced supported by international organisations that themselves are financing/promoting PPPs in education in LMICs (Verger et al., 2017). Interestingly, contrary to the prevalent view that PPP is cost‐effective means to promote education, a strand of the literature suggests that PPPs in education are in fact a costly activity where the taxpayers’ money is transferred to private business owners (Helby Petersen, 2019). The main concern is that PPPs in education are taking place for more than three decades, which requires updated consolidated evidence to guide future course of action for policy makers.

Previous systematic reviews have attempted to assess the impact of PPPs in education on access and quality mainly in LMICs. LaRocque (2008) and Patrinos et al. (2009) consolidated evidence on PPP in education before 2009. The review was updated by Snilstveit et al. (2015) by reviewing published literature till 2014. The trend of updating reviews continue and the latest available systematic reviews are by Aslam et al. (2017) and Crawfurd and Hares (2021). While these reviews provide useful information about the effectiveness of PPP in education, updating the evidence is always helpful for the policy makers to make the right decisions, especially when the world has witnessed a major disruption in the form of Covid19 pandemic. Therefore, these reviews need to be updated in terms of context, timeframe and focused outcomes of PPPs. Currently, no registered protocol is found regarding the effectiveness of PPPs in education in LMICs as evident in 3ie gap map. Building on Snilstviet et al (2015) and others, the proposed systematic review will focus on assessing the effectiveness of PPPs on both access and quality of education in LMICs. Additionally, our review will also expand the outcome variables. The ‘access to education’ is expanded to include enrolment, attendance, dropout, and completion, whereas the ‘quality of education’ is expanded to include both students’ learning outcomes and teachers’ pedagogical practices. This review will be a significant contribution to the existing literature and the ongoing developments on PPPs in education by bringing consolidated evidence from LMICs.

## 5 POLICY RELEVANCE

The review will contribute to evidence‐informed policy development in LMICs. The PPPs in education are now an integral part of educational policies in most of the LMICs but the questions related to their effectiveness are yet to be addressed with robust evidence. The findings of the review will stimulate policy and practice‐level discussions for improving school education through PPPs. Since international organisations and governments are promoting PPPs in education to bridge investment gaps and improve access, and quality of school education, the evidence emerging from this review will also help them make better policy decisions. We hope this review would help us identify gaps in evidence that can determine the extent to which the PPPs are effective in improving the access to and quality of education. It will further help in developing a research agenda for research on PPPs in education in the context of LMICs.

## 6 OBJECTIVES

This review intends to advance the work already done, mentioned above, with an emphasis on public‐private partnership as an intervention in education with more expanded inclusion criteria and its effectiveness for improving access and quality. The fundamental purpose of this review is to systematically consolidate, evaluate, and synthesize the available body of literature on the effects of PPPs on access to education (in terms of enrolment, attendance, dropout, and completion rates) and quality of education (in terms of students’ academic learning outcomes and teachers’ pedagogical quality). To do this, first, this review will examine and compare the impact of PPPs on access to education. Also, the review will compare students’ academic learning outcomes and teachers’ pedagogical quality in public, private, and PPP schools. Furthermore, it will examine the pattern and nature of empirical evidence in terms of geographical and methodological variations within available studies.

We acknowledge that not all PPPs are equal, the PPPs that include partnership between public and for‐profit‐private sector would differ significantly from the partnership between public and not‐for‐profit‐private sector. We also believe that it is important to examine the ‘mechanisms’ that could determine why particular PPPs work or do not work, and also the inequities that may arise through PPPs. However, we believe that once this systematic review finds out the impact of PPPs on improving access and quality of schools, we will be better able to attend to these very important concerns and aim to extend the review.

## 7 REVIEW QUESTIONS

**Major Question:** What is the impact of PPPs on improving access (i.e., enrolment, attendance, drop‐out, and completion) and quality (i.e., students’ learning outcomes and teachers’ performance) of primary and secondary schooling in LMICs?

**Subsidiary Questions:**

1. To what extent PPPs have increased access (i.e., enrolment, attendance, drop‐out, and completion) to education in LMICs compared to public and private schools?
2. To what extent do students from PPPs schools perform (i.e., students’ learning outcomes) differently than their counterparts in public and private school systems?
3. To what extent do teachers’ pedagogical quality in PPPs schools differ from their counterparts in public and private school systems?

## 8 METHODOLOGY

This review will follow Campbell systematic review guidelines (Shemilt et al., 2015). In this review, the empirical evidence on PPPs will be systematically collected, critically appraised to ensure the quality of methodology and reduce the biases, and analysed by employing meta‐analysis.

## 9 CRITERIA FOR INCLUDING STUDIES IN THIS REVIEW

Considering the objectives of this review, we will include studies that have evaluated the effectiveness of PPPs on educational access and quality in comparison to non‐PPP (i.e., public and/or private) schools. The included studies must be undertaken at the formal school level (primary and secondary) between 2000 and 2023 in the LMICs.

### 9.1 Types of studies

This review will include studies that have investigated the effectiveness of PPP schools on educational access and quality with no restriction of any specific model of PPP. Experimental studies including both randomised control trials and quasi‐experimental studies will be considered. However, qualitative evidence will be excluded because the intended outcomes cannot be measured qualitatively. This exclusion of qualitative studies does not necessarily indicate our disregard for this very important research tradition. It is just to limit our current work to a certain level and create some working boundaries. In our other work and some possible future work, we will continue to use both qualitative and quantitative traditions, wherever they are more useful. The focus of this review is to measure the effectiveness of PPPs in terms of increasing access and improving quality, which could best be determined using experimental design. In addition, studies that are available in the English language only will be included because none of the review team members understand other international languages.

### 9.2 Types of population

This review will include studies that have involved school students and teachers from primary and secondary grades who are part of PPP and public and/or private schools. It is important to note that the target population of all the selected studies must be students and/or teachers from primary to secondary level (grades 1−12). However, studies involving other types of schools such as early childhood education (preprimary), religious institutions, informal and nonformal will be excluded.

### 9.3 Types of settings

This review will include studies undertaken in any part of the low‐and‐middle‐income countries as defined by the World Bank. To be specific, we will include all three groups of countries of LMICs (i.e., upper middle‐income countries, lower middle‐income countries, and low‐income countries) aligned with categorisation of the World Bank.

### 9.4 Types of outcomes

This review will include studies that report the effectiveness of PPPs on any of the two key outcome variables: a) educational access with specific focus on student enrolment, attendance, drop‐out, and completion and b) quality with a focus on students’ learning outcomes and teachers’ teaching performance. However, we will exclude studies that have addressed outcomes other than our interest. For instance, we will not include outcomes targeting infrastructure and governance concerns at the PPP schools because these lack agreed benchmarks across the LMICs. In short, to be included, the studies must have evaluated the impact or effectiveness of PPPs at least on one of the educational outcomes listed below:

1. Enrolment: the total number of students enrolled in a school at the beginning of a specific grade year in the primary and secondary levels of school.
2. Attendance: the percentage of total school days on which enrolled students were present in an academic year.
3. Drop‐out: the number of students enrolled in school but did not attend or stopped to attend school throughout the year.
4. Completion: the number of students who have completed a particular grade level of school and/or a stage (primary and secondary) and/or full duration of schooling (grades 1‐12).
5. Students’ Learning Outcomes: the test results of students in school subjects (e.g., mathematics, science, languages).
6. Teachers’ pedagogical practices assessed through observation.

## 10 SEARCH STRATEGY

To facilitate this search, two groups of terms are created. The first category includes a glossary of words relating to PPPs in education because there are different ways of writing PPPs. The second category deals with quantified outcome variables, which can include concepts like educational access and educational quality. The outcome variables are further specified with their different search terms. By splitting the words in this way, it is intended to consider all the outcomes that might be pertinent while simultaneously excluding the substantial literature on PPPs in education related to its ideological, theoretical and policy basis. A Boolean “AND” and “OR” will be used to join these two groups of search terms.

The search terms and search query are decided by the review team with the consultation of a librarian. Three separate search queries are developed to retrieve the relevant studies on each targeted outcome variables. The table below presents three search queries for each review question.

**Table:** 

**Review question**	**Search query**
To what extent PPPs have increased access (i.e., enrolment, attendance, drop‐out, and completion) to education in LMICs?	(“public private partner*” OR “private public partner*” OR ppp*) AND (“enrolment” OR “attendance” OR “dropouts” OR “drop‐out” OR “completion”) AND (education) AND (school* OR Primary OR Elementary OR secondary)
To what extent do students from PPPs schools perform (i.e., students’ learning outcomes) differently than their counterparts in public and private school systems?	(“public private partner*” OR “private public partner*” OR ppp*) AND (“learning outcomes” OR “achievement” OR “test scores” OR “performance” OR “academic attainment” OR “grades” OR “results”) AND (education) AND (school* OR Primary OR Elementary OR secondary)
To what extent do teachers’ pedagogical quality in PPPs schools differ from their counterparts in public and private school systems?	(“public private partner*” OR “private public partner*” OR ppp*) AND (“teaching quality” OR “pedagogy” OR “pedagogical quality” OR “classroom practice” OR “teaching learning”) AND (education) AND (school* OR Primary OR Elementary OR secondary)

### 10.1 Search methods

We followed Campbell's information retrieval guidelines (i.e., Kugley et al., 2016) for literature searches. Before initiating a systematic review, it is important to undertake a rigorous search for existing systematic reviews (Glasziou et al., 2001). Therefore, a search for systematic reviews on PPPs will be conducted to identify relevant studies. However, the main search for the primary studies will be undertaken by the above mentioned three search queries. The electronic search will include various databases including general (Scopus and Campbell Library), subject‐specific (EBSCO Education Research Complete, NBER,), and grey literature (Dissertation and Theses Global – ProQuest and World Bank reports). We will not include conference proceedings. The electronic searches of the mentioned databases will be from 2000 to 2023. The search will filter results for English language only. The bibliographies from relevant studies will be checked to identify further studies missed by the initial search. Finally, we will also approach the scholars who are working on the PPPs in education to ensure that no important grey literature is missed.

## 11 DATA COLLECTION

In the stage of literature search two members from the review team will be involved in keeping the record of search query used in each database on which date and with the results retrieved. The search results from all databases will be imported into Covidence for literature screening. At first stage, two reviewers will independently scan the titles and abstracts using the above developed inclusion and exclusion criteria. At the second stage, two reviewers will conduct an independent full‐text review of the included studies using the same purpose‐designed eligibility criterion. Finally, the required information will be extracted from the included studies by two reviewers to ensure consistency and avoid missing out important data.

### 11.1 Quality assessment

After passing through the second stage, eligible studies will undergo an assessment of their quality. Since this review exclusively encompasses experimental studies, including randomized control trials and quasi‐experimental designs, an extensive process is necessary to evaluate the research procedures. All studies meeting the inclusion criteria will be subject to a methodological quality assessment by two independent reviewers using the Cochrane systematic reviews’ bias assessment tool known as RoB 2 (Risk of Bias 2). This tool will be adapted to suit the nature of the systematic review, which focuses on an educational intervention, specifically the PPPs (Public‐Private Partnerships in education). RoB 2 is a bias assessment tool developed and preferred by Cochrane systematic reviews, particularly for experimental studies. It assesses potential biases in both assignment and adherence that might influence the overall intervention outcomes. RoB 2 covers all five domains of risk of bias, which include the randomisation process, deviations from the intended intervention, missing outcome data, measurement of the outcome, and selection of the reported results. The tool comprises a series of signalling questions within each domain of risk of bias, a judgment criterion for the domain, which is cumulative of responses to each signalling question, a free‐text box for justifying the response, and an option to explain the direction of bias, such as low risk of bias (all criteria met), some concerns (one or more criteria are unclear), and high risk of bias (one or more criteria are not met).

### 11.2 Data extraction

The studies with no/low risk of bias will be included for data extraction. A standard extraction form will be developed for both randomised control trials and quasi‐experimental studies. Two independent reviewers will be oriented and trained for the process of data extraction. These two independent reviewers will extract the data from included studies. The data will include demographics of the included studies, information on study design, methods, study participants, and reported outcomes from both control and experimental groups with specific focus on sample size, mean and standard deviation. These statistics will be used for meta‐analysis. Following independent data extraction, a meeting will be held with co‐reviewers to resolve any discrepancies and obtain consensus. However, any unresolved disagreement will be referred to the core review team members.

## 12 DATA ANALYSIS

The extracted data will be used for meta‐analysis using RevMan software. The statistical data extracted (i.e., sample size, mean and standard deviation of both control and intervention groups) from included studies will be imported in RevMan in their respective study labels. A meta‐analysis of the outcome variables (access and quality) will be conducted separately by calculating the Standard Mean Difference effect size of each study on RevMan with a random‐effects model. The effect size with 95% confidence intervals will be reported, while Hedges’ g will be used to demonstrate the average effect of PPPs with sensitivity analysis such as heterogeneity (I^2^) not less than 90%, which is considered acceptable. The results will be presented using forest plots for each outcome variable separately.

## REVIEW AUTHORS

**Lead review author:** The lead author is the person who develops and co‐ordinates the review team, discusses and assigns roles for individual members of the review team, liaises with the editorial base and takes responsibility for the on‐going updates of the review.

## ROLES AND RESPONSIBILITIES

This is our review team composition.

- Content: Sajid Ali, Aisha Naz Ansari
- Systematic review methods: Aisha Naz Ansari, Sohail Ahmad
- Statistical analysis: Sadia Muzaffar Bhutta, Sohail Ahmad, Aisha Naz Ansari
- Information retrieval: Sohail Ahmad, Aisha Naz Ansari, Afaq Ahmed, Yasir Qadir

## POTENTIAL CONFLICT OF INTEREST STATEMENT

The conflict of interest form for all team members is attached with this application.

## PRELIMINARY TIMEFRAME

- Date you plan to submit a draft review: 31/07/2024
